# Man vs. machine: Predicting hospital bed demand from an emergency department

**DOI:** 10.1371/journal.pone.0237937

**Published:** 2020-08-27

**Authors:** Filipe Rissieri Lucini, Mateus Augusto dos Reis, Giovani José Caetano da Silveira, Flavio Sanson Fogliatto, Michel José Anzanello, Giordanna Guerra Andrioli, Rafael Nicolaidis, Rafael Coimbra Ferreira Beltrame, Jeruza Lavanholi Neyeloff, Beatriz D'Agord Schaan

**Affiliations:** 1 Department of Critical Care Medicine, Cumming School of Medicine, University of Calgary, Calgary, AB, Canada; 2 Data Intelligence for Health Lab, Cumming School of Medicine, University of Calgary, Calgary, AB, Canada; 3 Hospital de Clínicas de Porto Alegre, Universidade Federal do Rio Grande do Sul, Porto Alegre, RS, Brazil; 4 Department of Internal Medicine, Faculty of Medicine, Postgraduate Program in Medical Sciences: Endocrinology, Universidade Federal do Rio Grande do Sul, Porto Alegre, RS, Brazil; 5 Haskayne School of Business, University of Calgary, Calgary, AB, Canada; 6 Industrial Engineering Department, Universidade Federal do Rio Grande do Sul, Porto Alegre, RS, Brazil; Technion - Israel Institute of Technology, ISRAEL

## Abstract

**Background:**

The recent literature reports promising results from using intelligent systems to support decision making in healthcare operations. Using these systems may lead to improved diagnostic and treatment protocols and to predict hospital bed demand. Predicting hospital bed demand in emergency department (ED) attendances could help resource allocation and reduce pressure on busy hospitals. However, there is still limited knowledge on whether intelligent systems can operate as fully autonomous, user-independent systems.

**Objective:**

Compare the performance of a computer-based algorithm and humans in predicting hospital bed demand (admissions and discharges) based on the initial SOAP (Subjective, Objective, Assessment, Plan) records of the ED.

**Methods:**

This was a retrospective cohort study that compared the performance of humans and machines in predicting hospital bed demand from an ED. It considered electronic medical records (EMR) of 9030 patients (230 used as a testing set, and hence evaluated both by humans and by an algorithm, and 8800 used as a training set exclusively by the algorithm) who visited the ED of a tertiary care and teaching public hospital located in Porto Alegre, Brazil between January and December 2014. The machine role was played by Support Vector Machine Classifier and the human prediction was performed by four ED physicians. Predictions were compared in terms of sensitivity, specificity, accuracy, and area under the receiver operating characteristic curve (AUROC).

**Results:**

All graders achieved similar accuracies. The accuracy by AUROC for the testing set was 0.82 [95% confidence interval (CI) of 0.77–0.87], 0.80 (95% CI: 0.75–0.85), 0.76 (95% CI: 0.71–0.81) for novice physicians, machine, experienced physicians, respectively. Processing time per test EMR was 0.00812±0.0009 seconds. In contrast, novice physicians took on average 156.80 seconds per test EMR, while experienced physicians took on average 56.40 seconds per test EMR.

**Conclusions:**

Our data indicated that the system could predict patient admission or discharge states with 80% accuracy, which was similar the performance of novice and experienced physicians. These results suggested that the algorithm could operate as an autonomous and independent system to complete this task.

## Introduction

Overcrowding in emergency departments (EDs) is internationally recognized as one of the greatest challenges to healthcare provision [1]. Emergency department crowding is associated both with objective negative clinical endpoints, such as mortality [2], as well as flawed clinically important processes of care, such as lengthened time to treatment for patients with time-sensitive conditions [3]. Over the years, multiple small process improvement projects have attempted to improve ED overcrowding, but these processes do not improve the fundamental problem of improving hospital capacity [4]. Predicting ED attendances could help with resource allocation and reduce pressure on busy hospitals.

In healthcare operations, the growing use of intelligent systems (e.g. to support clinical decisions) may represent an important transition to a “new healthcare” [5]. Intelligent systems can help dealing with demands for more accurate diagnostics [6], safer and more effective medicines [7], and more effective treatments [8]. Using these systems may lead to improved diagnostic and treatment protocols, procedures [8] and help to predict hospital bed demand.

Emergency departments are time sensitive, highly stressful, non-deterministic, interruption-laden, and life-critical environments [9]. In these conditions, effective decision making is difficult or even impossible to achieve by humans. Despite some effort to develop intelligent systems to support ED decision-making [10], there is not much evidence from studies assessing their capability to operate as fully-automated, user-independent systems. We still lack knowledge on whether computer-based systems may be considered suitable to substitute humans in ED decision making, be it clinical or managerial decisions. This study targets such knowledge gap by investigating the performance of an intelligent system developed to predict hospitalizations and discharges, based on early ED patient text records using the SOAP (Subjective, Objective, Assessment, and Plan) framework comparing the performance of a computer-based algorithm and humans in predicting hospital bed demand in an ED.

## Methods

### Study database

This was a retrospective cohort study, which compared the performance of humans and machines in predicting hospital bed demand from an ED. Predictions were made by a set of physicians and by a trained algorithm, based solely on the records of the initial ED evaluation (i.e. SOAP notes), and compared to actual patient status after (admitted as an inpatient or discharged from the ED) 24 hours since first evaluation. This study was approved by the ethics committee of Hospital de Clínicas de Porto Alegre. Consent was not obtained because the data were analyzed anonymously and retrospectively. Authors have complied with the recommendations of the Declaration of Helsinki. The database was made available by the ED of an 842-bed, tertiary care and teaching public hospital located in the city of Porto Alegre, Brazil. Each electronic medical records (EMR) contained relevant information on early clinical care provided to patients, such as SOAP framework notes, prescriptions and exams. All textual records were written in Brazilian Portuguese.

EMR from all 16,703 patients who visited the ED between January and December 2014 were assessed in this study. Exclusion criteria were duplicated and empty textual records and those where patient final status were explicitly described in the SOAP notes; e.g. texts such as “HAA” (hospital admission authorized), “patient left”, “PD” (patient discharged), “patient not located”, and “patient did not answer when called”. Also excluded incomplete textual records, corresponding to those with missing information on one or more subjects of the SOAP framework. Consequently, the number of EMRs was reduced to 9,030, of which 4,673 were records from discharged patients and 4,357 were records of admitted patients; Table 1 details the reasons for records removal. Note that this hospital’s ED classifies patient acuity according to the Manchester Triage System [11], and is tasked within the local health system with providing care only to patients in the immediate (I), very urgent (VU), and urgent (U) categories. The balanced ratio between inpatient and discharged classes in the dataset reflects the type of care provided by the ED. No sample adjustments were made.

**Table 1:** Selection of reports from the database.

Description	Inpatient	Discharged	Total
Number of reports in the original database	8,038	8,665	16,703
Duplicated reports	-10	-5	-15
Information on final status explicit in reports	-191	-2,009	-2,200
Empty reports	-2,246	-1,067	-3,313
Incomplete SOAP reports	-1,234	-911	-2,145
Number of reports used in the study	4,357	4,673	9,030

SOAP: Subjective, Objective, Assessment, and Plan.

The database was adjusted to be used as input to both machine and human graders. Records were structured with two fields of information. The first was a dummy variable indicating patient final status (inpatient or discharged). The second contained the free-form textual SOAP notes entered by the hospital’s healthcare providers during the first patient-physician encounter.

Next, the dataset was randomly split into stratified (i.e. same ratio between classes, inpatient and discharged, as in the original sample distribution) training and testing sets. The training set was used to develop the machine’s algorithm and the testing set was used to compare machine and human graders. We sampled 2.5% of the records to comprise the testing set (230 records). This sample translates to a margin of error of 6.4%, with a confidence interval of 95%. The remaining 8,800 records were considered the training set. The ratio between records belonging to classes inpatient and discharged was kept constant in both sets, according to the original sample distribution. As a result, training and testing sets presented 4,553 and 120, and 4,247 and 110 records in classes discharged and inpatient, respectively.

### Intelligent system protocol

The machine role was played by Support Vector Machine Classifier (SVMC), which was the best predicting algorithm according to a previous analysis [12]. Textual information from all records were pre-processed and the algorithm trained. Next, the algorithm was used to predict the final status of 230 patients from the testing set.

The SVMC was first introduced by [13], being widely used to solve supervised classification problems from different application domains [14]. The two-class SVMC finds a hyperplane that ensures maximum separation between classes. Separating margins are identified by few data elements named support vectors [13]. The best predicting algorithm in [12] uses a variation of the two-classes SVMC called nu-Support Vector Classifier (nu-SVC). Proposed by [15], nu-SVC uses a parameter to control the number of support vectors and training errors. More details about the classifier are available in [15]. In this study, the nu-SVC was implemented using a linear kernel and default parameters of the package scikit-learn [16] from Python 3.6 [17].

As proposed previously [12], four pre-processing steps were carried out to prepare the textual information to be used as input to the algorithm: normalization, tokenization, feature selection, and conversion to set-of-words. In the normalization step, punctuation marks, numerical characters, and stop words were removed; capital letters were substituted by lowercases; and words were reduced to radical forms. In the tokenization step, the continuous string of characters of each record was broken down into linguistic units called tokens, which were delimited by blank spaces in the string. The algorithm uses the combination of unigrams, bigrams and trigrams as features, which are sequences of one, two or three adjacent words from the list of tokens. In the feature selection step, the F-Value of each feature from the training set was calculated. Following [12], features with an F-Value above the 65-th percentile of largest values were selected. The F-Value of a particular feature is calculated by:

$$F_{value(i)}=\frac{{\left(X_{i}^{imp}-X_{i}\right)}^{2}+{\left(X_{i}^{dis}-X_{i}\right)}^{2}}{\frac{1}{n_{imp}-1}\sum_{k=1}^{n_{imp}}{\left(X_{k,i}^{imp}-X_{i}^{imp}\right)}^{2}+\frac{1}{n_{dis}-1}\sum_{k=1}^{n_{dis}}{\left(X_{k,i}^{dis}-X_{i}^{dis}\right)}^{2}}$$ (1)

where *X_i_*, $X_{i}^{imp}$ and $X_{i}^{dis}$ is the average of the *i*-th feature in the complete, inpatient, and discharged datasets, respectively; $X_{k,i}^{imp}$ is the *i*-th feature of the *k*-th inpatient instance, and $X_{k,i}^{dis}$ is the *i*-th feature of *k*-th discharge instance. The last pre-processing step was the conversion to set-of-words representation, which results in a matrix indicating the occurrence of selected features in records. Matrix columns represent each of all selected features, while matrix rows represent each of the records. Matrix cells were filled out using term frequency–inverse document frequency (TF-IDF) indicator results. It may be expressed as:

$$TFIDF(t,d,D)=\frac{f(t,d)}{max\{f(t,d):t\in d\}}\times \log\frac{\left|D\right|}{\left|\left\{d\in D:t\in d\right\}\right|}$$ (2)

where *t* denotes the feature, *d* denotes the record, *D* is the total number of records in the collection, and *f*(*t,d*) is the number of occurrences of feature *t* in record *d*.

Once all pre-processing steps were performed, the algorithm was trained and the final status of patients from the testing set was predicted. Records of the testing set could be classified as belonging to one of two classes, inpatient or discharged. It was assumed that if a record was classified as inpatient, the patient stayed in the hospital and a ward bed was required. On the other hand, if a record was classified as discharged, there was no need for hospitalization. As a result, a list indicating the class of the 230 records in the testing set was made available. This list was used to measure the intelligent system performance in the present study.

### Human predictions

The manual prediction of the testing set was performed by four hospital’s ED physicians. Following [18], recruited physicians had different levels of experience. Two were novice ED physicians, with less than two years of experience working in the hospital’s ED, while the other two had been working in the hospital’s ED for more than 10 years.

Manual prediction was carried out in two steps. The first step aimed to verify substantial inter-observer agreement between physicians with similar experience. A trial testing subset of 30 randomized records was created, 14 belonging to the inpatient class and 16 belonging to the discharged class. All four physicians analysed the records and the Cohen’s Kappa coefficient [19] was used to measure their level of agreement. Kappa values between 0.61 and 0.80, and between 0.81 and 1.00 represent “substantial” and “almost perfect” strengths of agreement respectively [19]. Thus, 0.61 was used as minimum threshold for grouping evaluations from each pair of physicians with same experience.

The pair of novice physicians and the pair of experienced physicians disagreed in 4 and 5 predictions respectively. As a result, Cohen’s Kappa coefficient was 0.74 for novice physicians and 0.66 for experienced physicians; both scores indicated “substantial” level of agreement, surpassing the minimum established threshold for grouping evaluations.

In the second step, the remaining 200 records were split into subsets, observing class frequencies, and assigned to physicians for assessment. Eventually, each of the 200 records were analyzed by both experienced and novice physicians.

**Fig 1:**
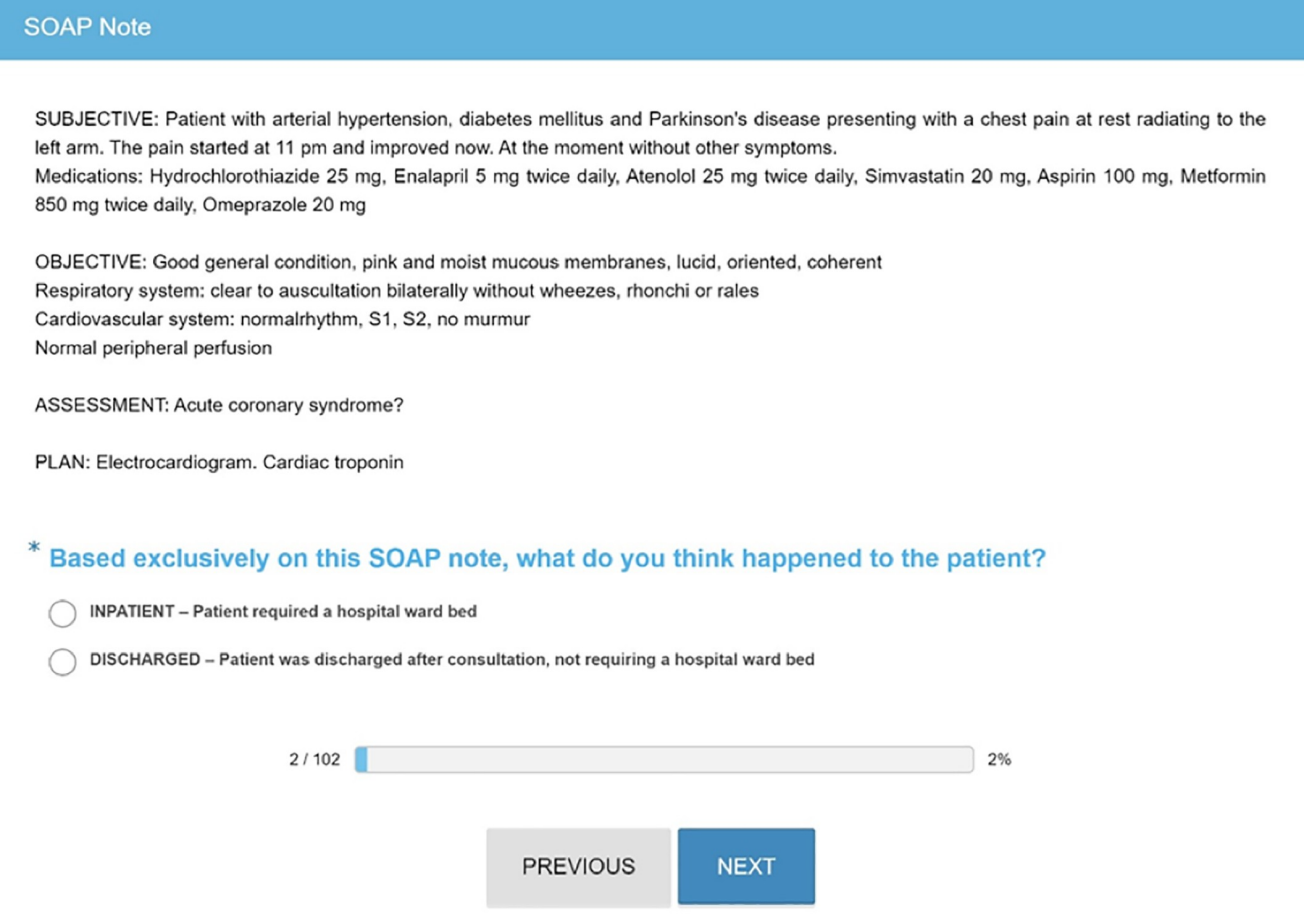
Record sample in SurveyMonkey (translated from Portuguese).

Physicians received personalized weblinks to access the records they were expected to evaluate. For each record, physicians were instructed to read and answer the following question: “Based exclusively on this SOAP note, what do you think happened to the patient?” Two options were given: (i) “INPATIENT–Patient required a hospital ward bed” or (ii) “DISCHARGED–Patient was discharged after consultation, not requiring a hospital ward bed”. Records consisted of four paragraphs, one to each subject of the SOAP framework. Physicians did not receive any feedback about their answers. In addition, physicians we not allowed to consult any other records, systems, colleagues, or other resources. Fig 1 shows an example of record presented to physicians.

In case of divergence between answers of physicians in the same experience group the conservative answer (“Inpatient”) was considered. The conservative answer maximized patient safety and was consistent with clinical practice when there is doubt regarding patient immediate prognosis.

### Statistical analysis

This study used validation methods similar to those in [18]. The statistical analysis was carried out in R x64 3.3.3 [20]. Receiver Operating Characteristic (ROC) analysis was performed for both machine and humans. Accuracies of the intelligent system protocol and human predictions were expressed as the area under the receiver operating characteristic curve (AUROC), and compared using the nonparametric test by [21]. Youden's J statistic [22] was used to calculate the optimal cutoff for sensitivity and specificity. According to [23], sensitivity and specificity are the same as true positive rate (i.e., the number of true inpatients divided by the total number of cases classified as inpatients) and true negative rate (i.e., the number of true discharged divided by the total number of cases classified as discharged), respectively. We considered a p-value equal to or smaller than 0.05 as indicative of significant differences.

## Results

All computations were performed on an Intel Core i7-7500U@2.9GHz and 16GB RAM. Machine training was completed successfully in 2 minutes and 46 seconds. Processing time per test EMR was 0.00812±0.0009 seconds. In contrast, novice physicians took on average 156.80 seconds per EMR, while experienced physicians took on average 56.40 seconds per EMR.

**Fig 2:**
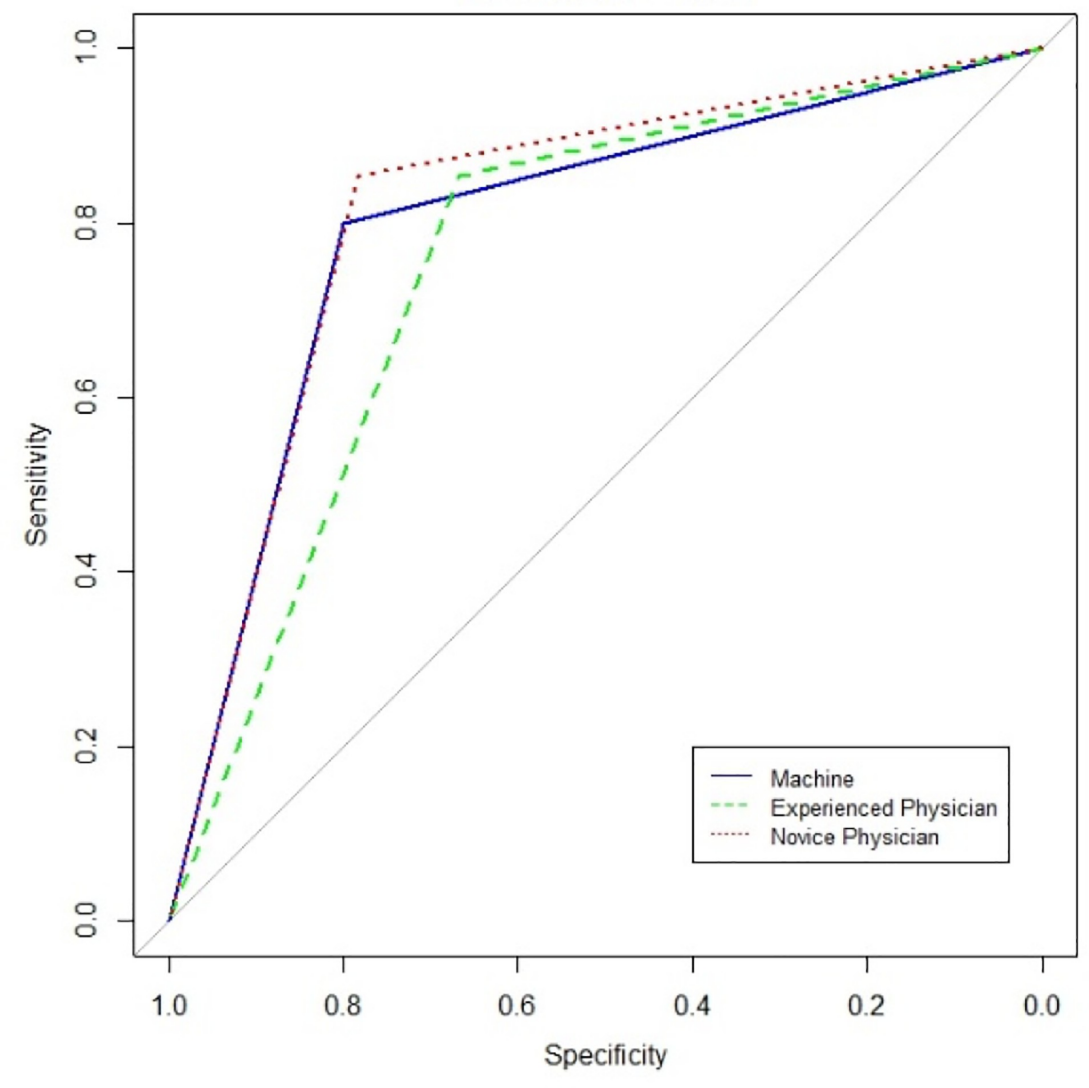
ROC curves by group.

Receiver Operating Characteristic curves for machine and novice and experienced physicians are presented in Fig 2.

Table 2 shows accuracy by AUROC for the testing set. All graders achieved similar accuracies. The accuracy by AUROC for the testing set was 0.82 [95% confidence interval (CI) of 0.77–0.87], 0.80 (95% CI: 0.75–0.85), 0.76 (95% CI: 0.71–0.81) for novice physicians, machine, experienced physicians, respectively. Table 2 also shows sensitivity and specificity for the testing set at optimal cutoff point.

**Table 2:** Accuracy, sensitivity and specificity with 95% CI considering the testing set.

	Machine	Novice Physicians	Experienced Physicians
Accuracy	0.80 (0.75–0.85)	0.82 (0.77–0.87)	0.76 (0.71–0.81)
Sensitivity	0.80 (0.73–0.87)	0.85 (0.78–0.92)	0.85 (0.79–0.92)
Specificity	0.80 (0.73–0.87)	0.78 (0.71–0.86)	0.67 (0.58–0.75)

Prediction accuracy by AUROC was not significantly different among graders (Table 3).

**Table 3:** DeLong’s test p-values for two correlated ROC curves.

Comparison	*p*-values
Machine *vs*. Novice Physicians	0.5528
Machine *vs*. Experienced Physicians	0.2395
Novice Physicians *vs*. Experienced Physicians	0.0517

Table 4 summarizes prediction success in the 230 cases. The machine was correct, but the physicians were not in 14 cases; the opposite occurred in 19 cases. All graders were correct in 137 cases, but incorrect in 10 cases. There were divergences between physicians in 50 cases.

**Table 4:** Comparison of mistakes and successes among graders.

Situation	Occurrences		
	Discharged Patients	Admitted Patients	Total
Machine and physicians were wrong	9 (3.91%)	1 (0.44%)	10 (4.35%)
Machine was correct and all physicians were wrong	12 (5.22%)	2 (0.87%)	14 (6.09%)
All physicians were correct and machine was wrong	10 (4.35%)	9 (3.91%)	19 (8.26%)
Divergences between novice and experienced physicians	23 (10.0%)	27 (11.7%)	50 (21.74%)
Machine and physicians were correct	66 (28.7%)	71 (30.8%)	137 (59.56%)
Number of reports used in the study	9 (3.91%)	1 (0.44%)	10 (4.35%)

The intelligent system protocol used 1,538,956 features to make predictions. The most informative features for admission were associated with the presence of symptoms (e.g. “edema”, “non-measured fev”, “paroxysmal nocturnal dyspnea”). On the other hand, the most informative features for discharge were related to normal physical examination or absence of symptoms (such as “well perfused extrem”, “normal breath sound”, and “no history dysuria”).

## Discussion

To our knowledge, this is the first study comparing an algorithm generated through machine learning with ED physicians in predicting patient admissions after their first evaluation. A computer running such algorithm could provide real-time data on bed necessity and aid bed management teams to improve patient flow processes. Our main results show that performances (accuracy) of physicians (novice or experienced) and machine were similar. Highest sensitivity was achieved by both novice and experienced physicians, and higher specificity was achieved by the machine, although confidence intervals had some overlap. In addition, the algorithm was significantly faster when compared to doctors. On average, novice and experienced physicians took 156.80 seconds and 56.40 seconds to analyze each record, respectively. In contrast, the algorithm analyzed the whole testing set in less than two seconds.

From a practical perspective, predictions made by the algorithm are almost instantaneous and allow immediate action from bed capacity planners, independent of the number of patients. There are some months in the year or times of the day that ED is most overcrowded as demonstrated by [24] using two scales: National Emergency Overcrowding Scale (NEDOCS), and Emergency Department Work Index (EDWIN). When humans are responsible for planning hospital bed demand, waiting times may increase when the ED is overcrowded, because of diminished physicians' availability.

In our study, the computer algorithm was as accurate as the doctors. Note that physicians reviewed records outside working hours in the ED, in a calm setting, without multitasking or time constraints. It is difficult to predict how their performances could be affected if evaluations were carried out in parallel with their ED working activities, but it is reasonable to speculate that they would be worse. On the other hand, machine performance is not affected by the working environment.

Improving patient flow and discharge processes through bed management supporting teams [25] have resulted in reduction in length of stay, cancelled interventions and increase in planned discharges [26]. The use of a computer algorithm as decision support system could enable hospital staff and health decision makers to better manage hospital inpatient beds, thus potentially reducing costs and inpatient length of stay [27]. Previous studies evaluated mathematical programs to model bed assignment [28] and prediction models to improve efficiency [29] with positive results, but none could made the predictions in real time, as it is possible with the herein tested algorithm.

The algorithm presented similar sensitivity and specificity values, while doctors tended to present higher sensitivity and lower specificity. That could be due to a clinical conservative bias–when in doubt, it would be safer to admit a patient then to discharge her. However, from a management standpoint a more balanced cut-off point on the ROC curve would be potentially more beneficial, since bed demand prediction would then be more stable with similar false negative and false positive rates. As [30] have discussed, a key requirement for effective bed management is information; anticipatory planning requires prediction of admissions and discharges. The authors have stated that there is a huge potential to improve information made available to bed management teams, and that few bed management functions have access to reliable data on patients expected to come in and be admitted.

The proportion of cases in which physicians were correct while the machine was wrong, and in which the machine was correct while physicians were wrong was similar (8.26% and 6.09%, respectively). The former group (physicians correct) is composed of nine admissions and ten discharges. On the discharges, it would seem that the algorithm has difficulty in recognizing information on patients being sent to other health services (two cases). On six other cases, there seemed to be extensive information on patient health history on the SOAP note, which was not directly related to the current condition of the patient. On the admissions, it is harder to presume causes for incorrect classification; for human readers, some SOAP notes clearly indicated either emergencies or the need for interventions: e.g. unstable angina, intestinal obstruction, nephrolithiasis necessitating a double J stent, and new onset atrial fibrillation. Since these descriptions were shortly stated on the Assessment item of the note, it is possible that the algorithm did not find enough information in the remaining of the note to justify admission and could not interpret the medical condition alone.

Regarding the cases where the algorithm was correct and physicians were wrong, most cases were discharges (12 of the 14 cases). This again may show a conservative bias from physicians, tending to classify cases as “admissions” when in doubt. It is hard for human readers to explain why the algorithm was correct; some SOAP notes describe serious diagnostic hypotheses, such as suspicion of acute coronary syndrome or of deep vein thrombosis, while others describe cases which appear to be non-complicated urinary tract infections, gastroenteritis or headaches. Human understanding of artificial intelligence (AI) decisions is known to be limited, and it has been proposed that it would be possible to train AI itself to provide natural language justifications of its decisions [31].

A possible refinement to the tested algorithm would be to include lab results into the available data; however, since the authors’ goal was to have the earliest possible prediction on patient admission or discharge, they chose to use only the first SOAP note registered by a physician. Indeed, the combination of four data mining operations including from lab results to clinical information let to a correct diagnosis in 98% of the cases in a pediatric emergency room, although retrospectively [32].

The study has some limitations. It was not confirmed whether the evaluators had the same clinical experience, the graduation time was used to choose the evaluators. The ED of HCPA provided care to high complexity cases, which includes only patients in the immediate, very urgent and urgent categories of the Manchester Triage System. The raw database presented a 50:50 proportion of cases in the inpatient and discharged categories. If the algorithm was applied to low complexity cases the results could be different. This is a single-hospital study, and thus its results may not be generalizable to other institutions. In one hand, few annotation studies assessed the SOAP framework for ED approaches and thus may be difficult to compare our results with other studies. No other machine learning options were explored, limited to the methods presented in the original document [12].

## Conclusion

The sophisticated computer algorithm here tested could predict patient admission or discharge with 80% accuracy, based solely on the first SOAP note from the ED. The algorithm had comparable performance to both novice and experienced ED physicians. We believe there are potential uses of intelligent computer systems in aiding hospital management, and it is possible that these systems could effectively support physicians in clinical decision making. The proposed algorithm is one such example; its implementation could provide useful data for bed management teams, improving patient flow processes throughout the hospital.
